# A Nanoscale Interface Promoting Molecular and Functional Differentiation of Neural Cells

**DOI:** 10.1038/srep31226

**Published:** 2016-08-09

**Authors:** Tamara Posati, Assunta Pistone, Emanuela Saracino, Francesco Formaggio, Maria Grazia Mola, Elisabetta Troni, Anna Sagnella, Morena Nocchetti, Marianna Barbalinardo, Francesco Valle, Simone Bonetti, Marco Caprini, Grazia Paola Nicchia, Roberto Zamboni, Michele Muccini, Valentina Benfenati

**Affiliations:** 1 Consiglio Nazionale delle Ricerche (CNR), Istituto per lo Sintesi Organica e la Fotoreattività (ISOF), via Gobetti, 101, 40129, Bologna, Italy; 2 Consiglio Nazionale delle Ricerche (CNR), Istituto per lo Studio dei Materiali Nanostrutturati (ISMN), via Gobetti, 101, 40129, Bologna, Italy; 3 Dipartimento di Bioscienze, Biotecnologie e Biofarmaceutica, Università degli Studi di Bari “Aldo Moro”, Via Amendola 165/A, 70126, Bari, Italy; 4 Laboratorio di Micro e Submicro Tecnologie abilitanti dell’Emilia-Romagna (MIST E-R), Via P. Gobetti 101, I-40129 Bologna, Italy; 5 Dipartimento di Scienze Farmaceutiche, University of Perugia, Via del Liceo 1, 06123, Perugia, Italy; 6 Department of Pharmacy and Biotechnology, via S. Donato 19/2, University of Bologna, 40127 Bologna, Italy

## Abstract

Potassium channels and aquaporins expressed by astrocytes are key players in the maintenance of cerebral homeostasis and in brain pathophysiologies. One major challenge in the study of astrocyte membrane channels *in vitro,* is that their expression pattern does not resemble the one observed *in vivo*. Nanostructured interfaces represent a significant resource to control the cellular behaviour and functionalities at micro and nanoscale as well as to generate novel and more reliable models to study astrocytes *in vitro*. However, the potential of nanotechnologies in the manipulation of astrocytes ion channels and aquaporins has never been previously reported. Hydrotalcite-like compounds (HTlc) are layered materials with increasing potential as biocompatible nanoscale interface. Here, we evaluate the effect of the interaction of HTlc nanoparticles films with primary rat neocortical astrocytes. We show that HTlc films are biocompatible and do not promote gliotic reaction, while favouring astrocytes differentiation by induction of F-actin fibre alignment and vinculin polarization. Western Blot, Immunofluorescence and patch-clamp revealed that differentiation was accompanied by molecular and functional up-regulation of both inward rectifying potassium channel Kir 4.1 and aquaporin 4, AQP4. The reported results pave the way to engineering novel *in vitro* models to study astrocytes in a *in vivo* like condition.

The capability of the Central Nervous System (CNS) to receive, integrate and compute information from and to the body is dependent on the maintenance of an electrochemical gradient of ions, organic molecules and osmotically driven water across the plasma-membrane of neural cells. Astrocytes, by contacting neurons and cells lining the fluid-filled compartments (microvasculature, ventricular and subarachnoid spaces) are strategic players in the homeostatic regulation of the brain. They are endowed with ion and water transmembrane channel proteins that are localized in specific plasma-membrane microdomains facing diverse liquid spaces. The astroglia-mediated homeostasis of the CNS *in vivo* relies on the maintenance of their morphology as well as the activity and distribution pattern of protein channels belonging to different microdomains^1^,^2^,^3^. Notably, derangement of ion and volume homeostasis can lead to pathophysiologies, such as hypoxia/ischemia, stroke, different forms of epilepsy and Alzheimer disease^4^,^5^,^6^, which are characterized by perturbation of the astrocytic morphology and alterations to microdomain protein surface expression and/or polarized localization.

Among different types of voltage-dependent ion channels expressed by astrocytes *in vitro* and *in vivo*^7^,^8^, the inward rectifying K^+^ conductance, mediated by the Kir4.1 channel, is mainly involved in the astroglial control of extracellular K^+^ homeostasis^9^,^10^. Notably, in the sclerotic hippocampus of patients with medial temporal lobe epilepsy, loss or disruption of the microdomain protein complex anchoring Kir4.1 is thought to alter K^+^ buffering and clearance, thus contributing to the epileptiform activity^11^,^12^,^13^. Expression of Kir 4.1 mRNA and protein, as well as the Kir 4.1 immunoreactivity increases markedly with increasing pathologic grade of human astrocytic tumors^14^,^15^.

Studies *in situ* and *in vivo*, have identified aquaporin 4 (AQP4) as the molecular partner of Kir4.1 in the astroglial microdomains involved in the clearance of K^+^, by facilitating osmotic water movement through the plasma membrane^16^,^17^. It has been established that AQP4 is the first molecular player involved in pathological brain swelling leading to cytotoxic edema^18^,^19^. In primary brain tumors such as gliomas, K^+^ conductance and AQPs are potentially involved in mechanisms that promote cell invasion and the formation of brain metastasis^18^,^19^,^20^,^21^. In a mouse model of Alzheimer Disease (Tg-ArcSwe mouse) changes in AQP4 expression are evident at the early stages of plaque formation, indicating that a loss of astrocytic AQP4 polarization could play a key role in the pathogenesis of the disease^6^.

All these pieces of evidence indicate that generating models intended for studying, manipulating and controlling of astrocytes Kir4.1 and AQP4 activity are therefore highly desirable.

When astrocytes are grown in culture, they lose their characteristic star-like shape as well as their AQP4 and Kir4.1 plasma membrane localization observed *in vivo*^2^,^22^,^23^,^24^,^25^.

In this context, increasing attention is being paid to novel models, materials, nanostructured interfaces and devices enabling the control and modulation of protein channel serving micro-domain functionalities in astrocytes that might help to generate significant knowledge in CNS physiology and pathophysiology^22^,^23^,^24^,^25^,^26^,^27^,^28^,^29^,^30^,^31^. In particular, nano-engineered materials and devices have been shown to interact with neural cells, offering unprecedented potential to control the morphology and calcium signalling of astroglial cells at the nanoscale^23^,^26^,^27^,^28^,^29^,^30^,^31^,^32^. However, at present, nanomaterials capable of inducing astrocyte AQP4 and Kir4.1 polarized expression and modulation of their function *in vitro* have not yet been reported.

Hydrotalcite-like compounds (HTlc) also known as “anionic clays” or layered double hydroxides (LDHs), are a particular family of layered materials consisting of positively charged layers and exchangeable interlayer anions to maintain overall charge neutrality. The great scientific and technological relevance of HTlc stems from their tuneable layer charge density, variable elemental composition, high chemical stability and rich intercalation chemistry^33^,^34^,^35^. These advantages, together with their low toxicity and biocompatibility^36^,^37^, make HTlc a very attractive class of layered solids, which find application in different fields such as medical science^38^,^39^,^40^,^41^,^42^,^43^,^44^,^45^, polymeric nanocomposites^46^,^47^ as well as photonics, sensing and opto-electronic devices^48^,^49^,^50^. Interestingly, it has been shown recently that thin films characterized by “rod-like” structures can be obtained from aqueous colloidal dispersion of ZnAl-HTlc nanoparticles^51^.

Here, we evaluate the effect of the interaction of HTlc nanoparticle films with primary cultured rat neocortical astrocytes. We provide molecular and functional clues that HTlc films are suitable substrates to generate novel *in vitro* models to study the specific functionality and expression of astrocyte membrane channels Kir4.1 and AQP4.

Cell viability assays and morphological analysis here demonstrate the biocompatibility of HTlc films. The study of cells/substrate interaction indicates that HTlc films promotes astrocyte differentiation and favours a starlike morphology, more typical of astrocytes observed *in vivo*. The effect cannot be attributed to a gliotic reaction, as underlined by the absence of upregulation of Glial Fibrillary Acid Protein (GFAP), a typical marker of astrogliosis. The rearrangement of actin cytoskeleton and of vinculin focal adhesion point is involved in the observed effect. Molecular and functional analyses reveal that differentiation is accompanied by up-regulation in the expression and function of both inward rectifying potassium channel Kir 4.1 and AQP4.

These results pave the way for the potential use of HTlc films as new nanointerfaces targeted to modulation, recovery and study of astroglial functionalities and pathophysiologies *in vitro*.

## Results and Discussion

### Dropcasted HTlc films have nanoscale, rod-like structures and form an optically transparent substrate

**Figure 1:**
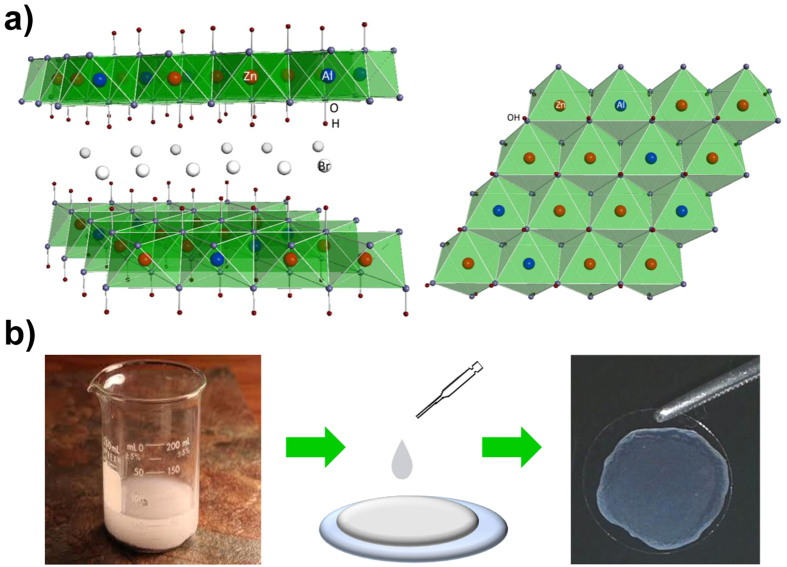
Structure and preparation of ZnAl-HTlc films. (**a**) Schematic representation of the side view (left) and of the top view (right) of the ZnAl-HTlc structure. (**b**)Scheme showing ZnAl-HTlc film preparation.

**Figure 2:**
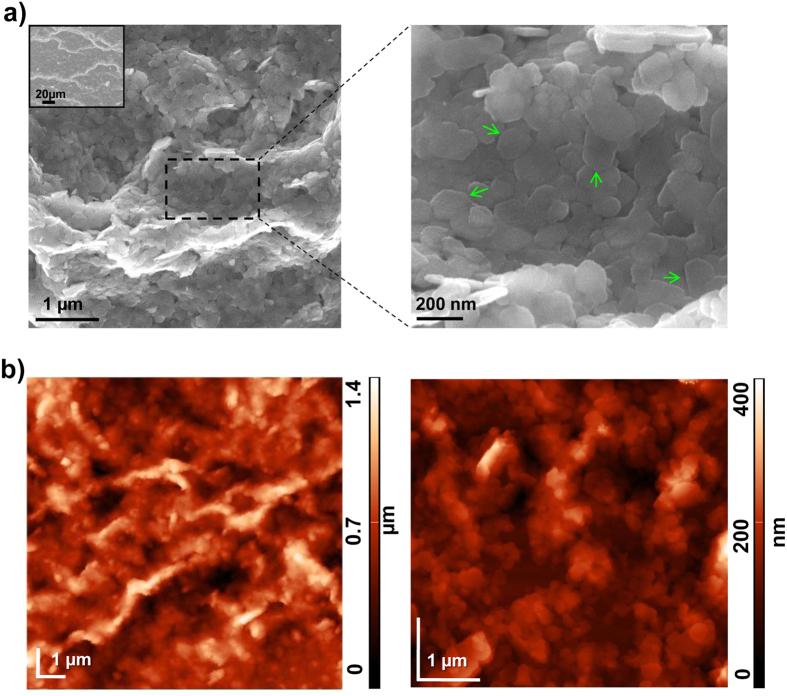
Morphological characterization of HTlc films. (**a**) SEM images at different magnifications of ZnAl-HTlc film (**b**) AFM topographical images of ZnAl-HTlc film (RMS~134 nm).

To fabricate an easy to use nanoscale biocompatible interface we sought to take advantage of the self-assembly capability of HTlc^49^,^50^,^51^,^52^,^53^,^54^. Specifically, we used HTlc having the formula [Zn_0.72_Al_0.28_(OH)_2_]Br_0.28_ 0.69 H_2_O, the chemical structure of which is schematically reported in Fig. 1a. An aliquot of the reported HTlc colloidal aqueous dispersion, at a concentration of 1 mg/mL, was dropped onto a glass coverslip and successively dried in a sterile hood (Fig. 1b). Scanning electron microscopy (SEM) and atomic force microscopy (AFM) imaging were performed to provide an insight into the surface morphology, organization and roughness of HTlc films. SEM images at different magnifications of HTlc samples are reported in Fig. 2a. As previously observed^51^,^52^, the colloidal aqueous dispersion of HTlc having nanometric dimensions have a very high tendency to spontaneously form films. The formation of dense stacks of platelet-like HTlc nanocrystals results in rod-like structures in which individual nanocrystals aggregate in a preferential direction (Fig. 2a right panel and inset). This behaviour can be related to the method used for film fabrication: small platelets of hydrotalcite in water probably migrate to the surface of the aqueous dispersion droplet during the slow solvent evaporation and aggregate assuming the rod-like shape^51^. At higher magnification, the typical hexagonal shape (see green arrows in Fig. 2a, left panel) and the dimensions (diameter of approximately 150–200 nm) of the HTlc nanoplatelets can be observed according to previous literature data^51^. AFM images of HTlc films revealed the typical rod-like structure, in agreement with SEM observations, and a surface roughness (RMS) of approximately 134 nm (Fig. 2b). Transparency is an essential feature for nanostructured interfaces to be used for *in vitro* cell culture. The transparency to visible light of HTlc films was investigated by light transmittance measurements. Supplementary Figure S1a shows a transmission of 43–78% for HTlc across the visible spectrum (300–800 nm). The fair visible-light transparency of HTlc films can be attributed to the flat and uniform orientation of the HTlc platelets in the films. Figure S1b shows the X-ray diffraction (XRD) patterns of HTlc films and HTlc nanoparticles in powder form. The interlayer distance (8.06 Å) of the HTlc in the films matches that of the HTlc powder and is compatible with the presence of bromide anions in the interlayer region. It is noteworthy that in the HTlc films, the intense 00l reflections and the absence of any in-plane reflections (h, k ≠ 0) at high angles is evidence of an extremely well c-oriented assembly of HTlc nanoplatelets^49^,^53^,^54^.

### HTlc nanoparticle films promote astrocyte morphological differentiation

In order to determine the impact of HTlc substrate on astroglial cell growth, pure rat neocortical astroglial cells were re-plated on glass coverslips coated with poly-D-Lysine (PDL) or with films obtained by casting a dispersion of HTlc nanoparticles.

**Figure 3:**
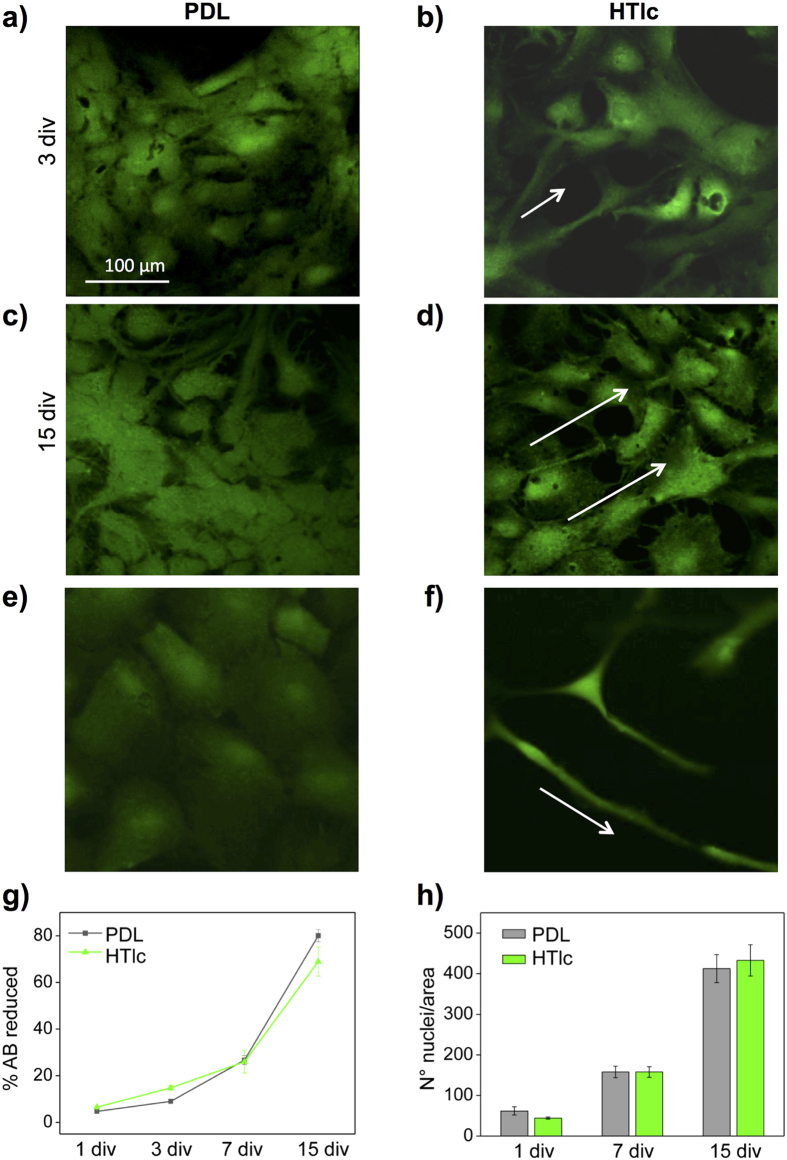
Viability and morphology of astrocytes on PDL and HTlc films. (**a–f**) Single plane confocal images of astrocytes stained for FDA, representing viable cells plated on PDL (**a,c**) and on HTlc (**b,d**) captured after 3 div (**a,b**), and 15 div (**c,d**). (**e,f**) Low density plated astrocytes captured after 7 div; note differentiated astrocytes plated on HTlc (**g**) Time course of astrocyte viability on PDL and HTLc film, investigated by AB assay at different time points. Data are plotted as the averaged percentages of reduced AB ± Standard Error (SE) versus div. (**h**) Histogram plot reporting the averages of astrocyte nuclei number, counted on images of cells grown on PDL (gray bars) and on HTlc films (green bars). (p > 0.05, independent t test).

Fluorescein diacetate (FDA) viability assays were performed after 3 days *in vitro* (div), 7 div and 15 div from re-plating astrocytes. Single plane confocal imaging analyses and morphological observations after 3 div and 15 div, reported in Fig. 3, revealed viable astrocytes plated on PDL (Fig. 3a,c) and HTlc films (Fig. 3b,d). Polygonal flat shapes, resembling the typical phenotype of *in vitro* primary astrocytes grown on poly-styrene Petri dishes and silk biomaterials^22^,^23^,^24^,^25^ were observed in cells cultured on PDL film. This morphology is retained until confluence that is reached after 15 div (Fig. 3d). After 3 div from replating, astrocytes plated on HTlc films displayed a differentiated phenotype with processes departing from the cell body, which projected endfeet on distal cells (Fig. 3b, white arrows). Moreover, after two weeks of growth, the distribution of cells on HTlc was not uniform but followed a specific linear pattern (Fig. 3d, white arrows). The effect was even more relevant on cells plated at low density (Fig. 3e,f). Quantitative analysis of cell viability was obtained by AlamarBlue (AB) assay. As shown in Fig. 3 (Fig. 3g), the metabolic activity of astrocytes plated on HTlc increased over time with a rate comparable to that of cells grown on the PDL substrate. To verify astrocyte adhesion and proliferation on HTlc substrate, images of cells stained with DAPI were taken after 1 div, 7 div and 15 div (Supplementary Figure S2) and the cell nuclei in each image were counted. In agreement with the cell viability assay, the histogram plot of the DAPI stained cell nuclei number/area, counted at different time points (Fig. 3h) indicated that the number of astrocytes on HTlc samples was comparable to that on PDL. (Supplementary Figure S2). It should be noted that long-term survival of astrocytes was severely compromised (Supplementary Figure S3a) when substrates were made from micrometric HTlc (Figure S3b).

The percentage of differentiated astrocytes was determined in cells plated on PDL and HTlc by measuring the ratio between the vertical and horizontal axis of each cell with an elongated and star-like morphology (see experimental section). Quantitative analyses confirmed the observation that the percentage of differentiated astrocytes was higher when cells were grown on HTlc after 3 div compared to those grown on PDL. The difference was even more relevant after 7 div where the number of differentiated cells was 5 times higher on HTlc compared to the PDL value (Table 1). We also quantified the number of cell protrusions in astrocytes displaying radial and lateral processes. The number of protrusions is expressed as a percentage of the total number of differentiated cells and was classified as: “= 2 ” and “> 2 *”.* After 3 div and 7 div 80 ± 0.6% % and 88.6 ± 0.7%, respectively, of differentiated cells displayed only 2 long protrusions while the minority displayed from 3 to 6 process. These data indicate that the elongated morphology was more evident than the stellated one in HTlc plated astrocytes.

**Figure 4:**
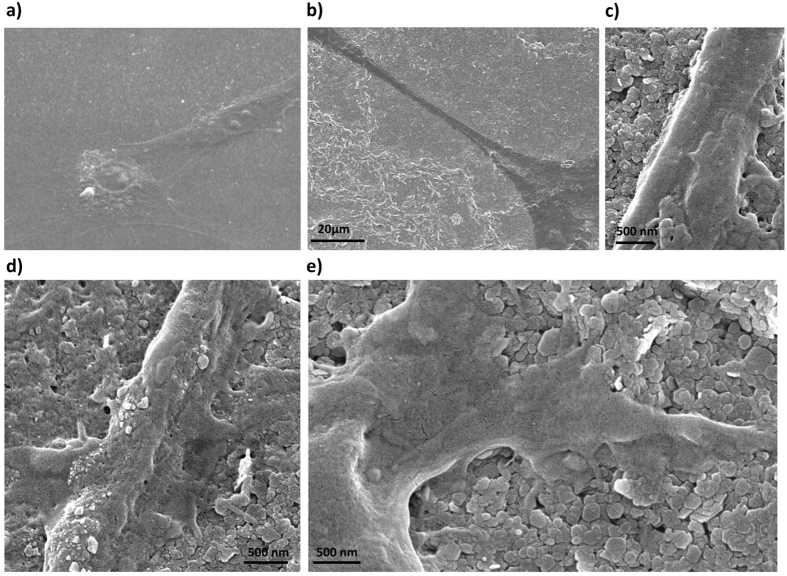
SEM imaging of cells plated on PDL (**a**) and HTlc (**b–e**): note the presence of elongated branches in astrocytes plated on HTlc that follow the rod like structure of HTlc. (**c**–**e)** high magnification images of cells plated on HTlc films revealing lateral endfeet in astrocyte processes projecting to HTlc nanoplatelets.

To gain further insight into the effect of HTlc film morphology on astrocyte differentiation, we performed SEM images after 5 div from re-plating of astrocytes grown on PDL and HTlc (Fig. 4a,b, respectively). Accordingly the cell morphology on PDL substrate resembled the polygonal shape reported in Fig. 2 (Fig. 4a). On the other hand with respect to PDL, SEM images of astrocytes plated on HTlc films revealed that astroglial branches can be seen near rod-like structures at an average distance of 10 ± 1μm (Fig. 4b, n of images = 30). Please note, images with higher magnification (Fig. 4c–e) revealed that astrocytes were sealed to HTlc films by lateral small endfeet projections anchored to HTlc nanoplateles.

The results on morphology and cell survival analyses indicate that HTlc does not promote proliferative or hypertrophic cell growth, typical for astrogliotic cells (Fig. 3)^55^,^56^. On the contrary, they suggest that HTlc film promotes the morphological phenotype of astrocytes, typically observed in differentiated state occurring,for example, upon long term treatment with dibutyrril-cAMP^24^. It is also worth noting that process extension and cell growth following a specific direction pattern was observed on HTlc films (Fig. 4). The formation of rod-like structure evidenced in Fig. 1 on HTlc samples could in fact induce the reported effect. We observed that astroglial cell viability was severely compromised over the long term when substrates were made of HTlc microparticles (Figure S3a), demonstrating the importance of the substrate dimensions and nanostructure for cell adhesion and viability. In this regard, it should be noted that films cannot be obtained by casting a suspension of micrometric HTlc (Figure S2b). Thus, detachment of HTlc powder from the substrate to the solution occurring over a long term could account for the lower cell viability.

Previous studies have shown that coating a pure Mg surface with Mg–Fe HTlc led to better cell adhesion and growth of human mesenchymal stem cells^57^. However, our study is the first to investigate the biocompatibility of HTlc films with cells of the CNS. Indeed, previously works reporting on biocompatibility with PC12 and cortical primary neurons^58^, referred to nanoparticle suspension and not to HTlc films.

### HTlc nano-particle films promote astrocytes morphological differentiation by promoting cytoskeleton rearrangement

**Figure 5:**
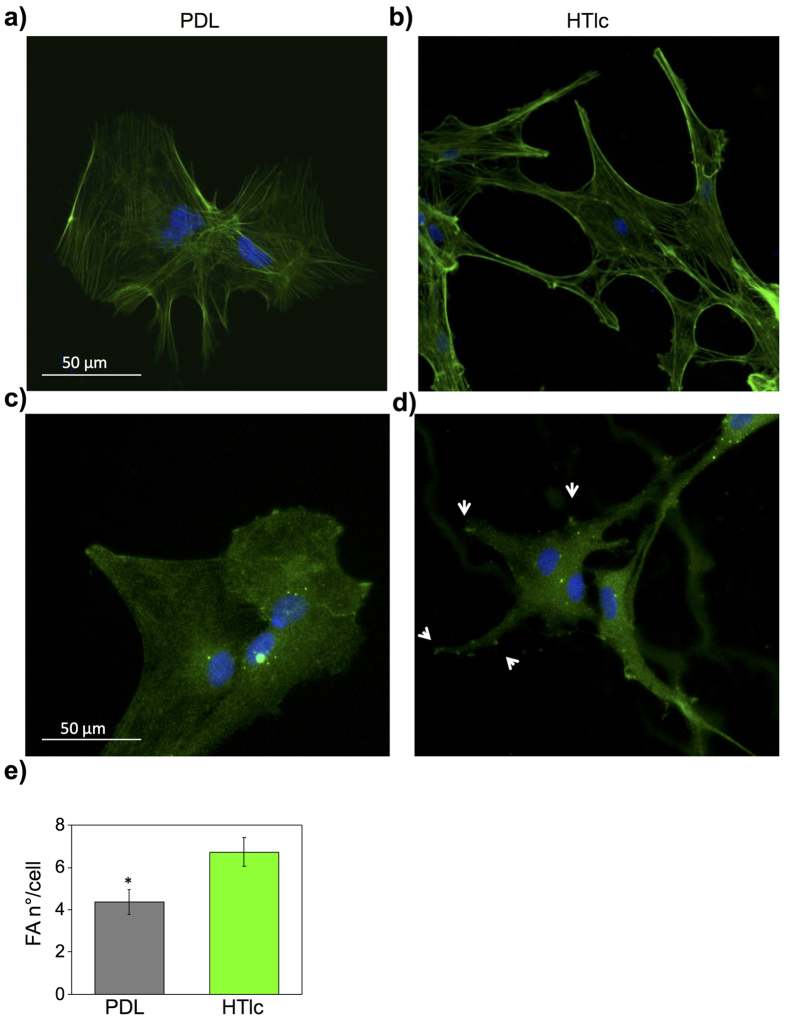
F-actin cytoskeleton and Vinculin proteins expression in astrocytes plated on PDL and HTlc. Micrographs representing astrocytes imunostained for actin (**a,b**) and vinculin (**c,d**) grown on PDL (**a**,**c**) and HTlc fims (**b,d**). (**e**) Hstogram plot reporting the number of Focal Adhesion (FA) contacts per astrocyte, which were counted in cells plated on PDL (gray bars) and on HTlc films (green bars). Data are expressed as means ± SE (n = 25; *p < 0.05).

In order to investigate the mechanisms underpinning astrocytes and HTlc interaction, we performed F-actin and vinculin staining to mark cytoskeletal fibres and focal adhesion complex. As shown in Fig. 5 and Supplementary S4, randomly interweaved F-actin fibres were observed on cells grown on PDL (Fig. 5a and Figure S4). A more ordinate and parallel pattern of actin fibres was observed in cells grown on HTlc films (Fig. 5b and Figure S4) with evident stressed fibres and a pronounced expression of actin on lateral endfeet. Astrocytes plated on PDL showed a mislocalized vinculin signal (Fig. 5c), while focal adhesion tips appeared to be more localized in the endfeet and branches of astrocytes grown on HTlc films (Fig. 5d arrowhead). We also quantified Focal Adhesion points (FA) by counting the number of vinculin contacts per cell (Fig. 5e). The histogram plot of the averaged FA number/cell revealed a significantly higher rate of FA contact on HTlc with respect to the PDL control (p < 0.05). Collectively these data support the tenet that HTlc films induce differentiation in astrocytes by promoting cytoskeleton and focal adhesion point rearrangement.

The reported results are in agreement with previous studies indicating that primary rat astrocyte differentiation can be obtained by treatment with agents that induce rearrangement of the cell cytoskeleton^59^. Moreover, it has been shown that nano- and micro-scale structured substrates induce stem cell and glial cell differentiation by activating mechano-transduction pathways which are initiated by integrin adhesion to the substrates that in turn initiate the rearrangement of the cell cytoskeleton^60^. Thus the effect we observed is likely to be due to the interaction of the astrocyte cell membranes with rod-like structures formed in HTlc film nanoplateles (see Fig. 4b). In particular we focused on vinculin that is a component of the membrane-associated adhesion complexes that bind cells to extracellular matrix and adjacent cells. Vinculin is involved in regulating many aspects of cell-matrix adhesion, including the assembly, turnover and strength of focal adhesions, as well as tuning polymerization and modifying the structure of existing actin filaments^61^. Moreover, it has been demonstrated that morphological changes to astrocytes are accompanied by vinculin rearrangement^62^. In this view our data suggest that the vinculin rearrangement we observed is likely to be involved in initiation and induction mechanisms of astroglial differentiation induced by HTlc films.

**Figure 6:**
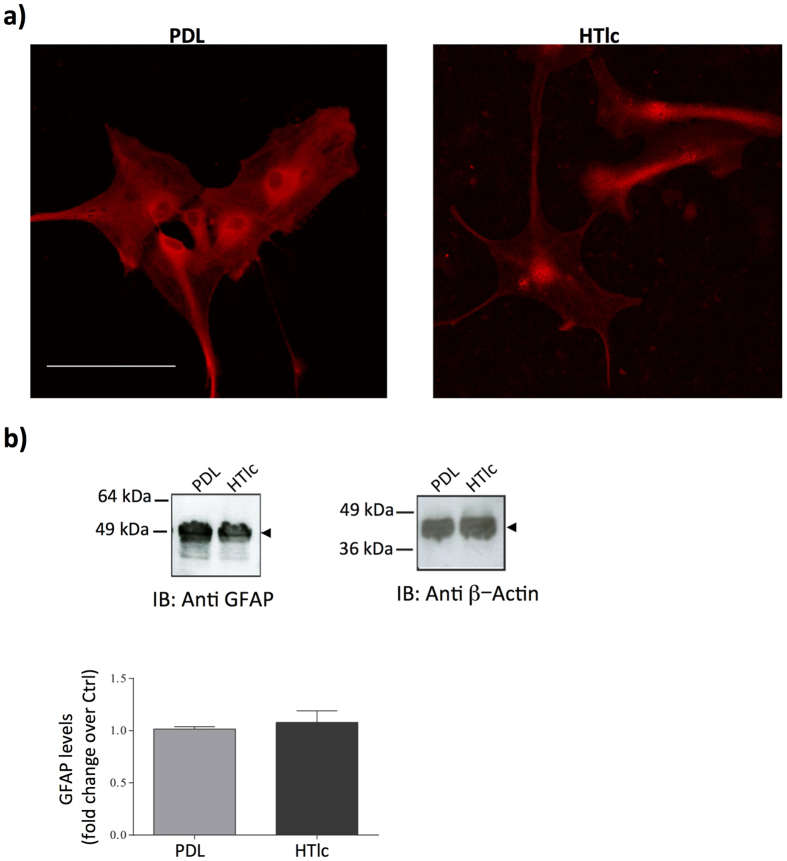
GFAP protein expression in astrocytes plated on PDL and HTlc. (**a**) Confocal imaging of astrocytes stained with GFAP. Scale bar is 50 μm. (**b**) immunoblotting and quantification of the expression level of GFAP. β-actin (right panel) is used as control for signal normalization. Lower panel: histogram plots evidencing protein fold of change over the control. Note that the expression level of GFAP was not significantly different between PDL and HTlc (n = 3). Cropped images of the entire blots are reported. However, no other specific signals were observed within the blot length.

Since morphological alteration occurs upon gliosis^55^,^56^, we next evaluated the distribution and expression level of GFAP, a well known marker of astrogliotic cells *in vivo* and *in vitro,* by performing confocal imaging of immunofluorescent staining (Fig. 6a) and immunoblotting (Fig. 6b) of GFAP in astrocytes grown on PDL and on HTlc film, after 5 div. Immunofluorescence and immunoblotting confirmed that the expression levels of GFAP were not different in cells grown on PDL and HTlc, thereby indicating that HTlc does not promote gliotic reactions *in vitro*.

### HTlc films promote molecular and functional expression of AQP4 and Kir4.1 on the cell membrane

We then investigated the effect of HTlc on ion channels and AQP4, relevant for astroglial homeostasis. With this in mind, we performed immunofluoresence and confocal analyses to determine the expression of Kir 4.1 and the AQP4 distribution patterns on PDL and HTlc plated astrocytes.

**Figure 7:**
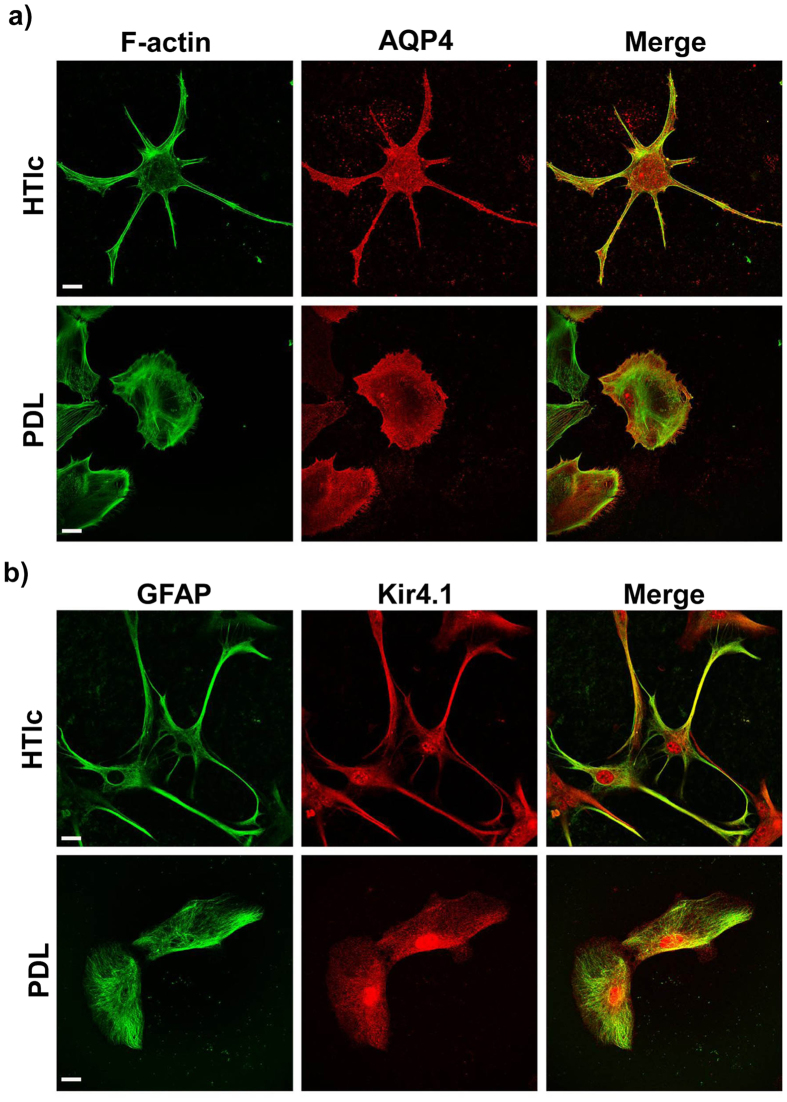
AQP4 and Kir 4.1 protein expression in astrocytes plated on PDL and HTlc. (**a)** Confocal imaging of astrocytes grown on HTLc NPs (upper panels) and PDL (lower panels) and stained for AQP4 (red), and Actin (green). (**b)** Confocal imaging of astrocytes grown on HTLc NPs (upper panels) and PDL (lower panels) and stained for Kir4.1 (red), and GFAP(green). A merged image of the channels is reported on the right side of the panel, revealing that AQP4 and Kir 4.1 are highly up-regulated in the endfeet of astrocytes grown on HTlc NPs.

Typical single plane confocal imaging of astrocytes grown on PDL and HTlc stained for AQP4 (red) Kir4.1 (green) and F-actin (blue) are reported in Fig. 7. Importantly, while the expression of AQP4 and Kir4.1 in cells grown on PDL was mislocalized, cells with differentiated morphology grown on HTlc displayed a marked expression of both protein in the plasma membrane of astroglial endfeet and a co-localization with actin fibres in the proximity of the cell membrane (Fig. 7, merge).

**Figure 8:**
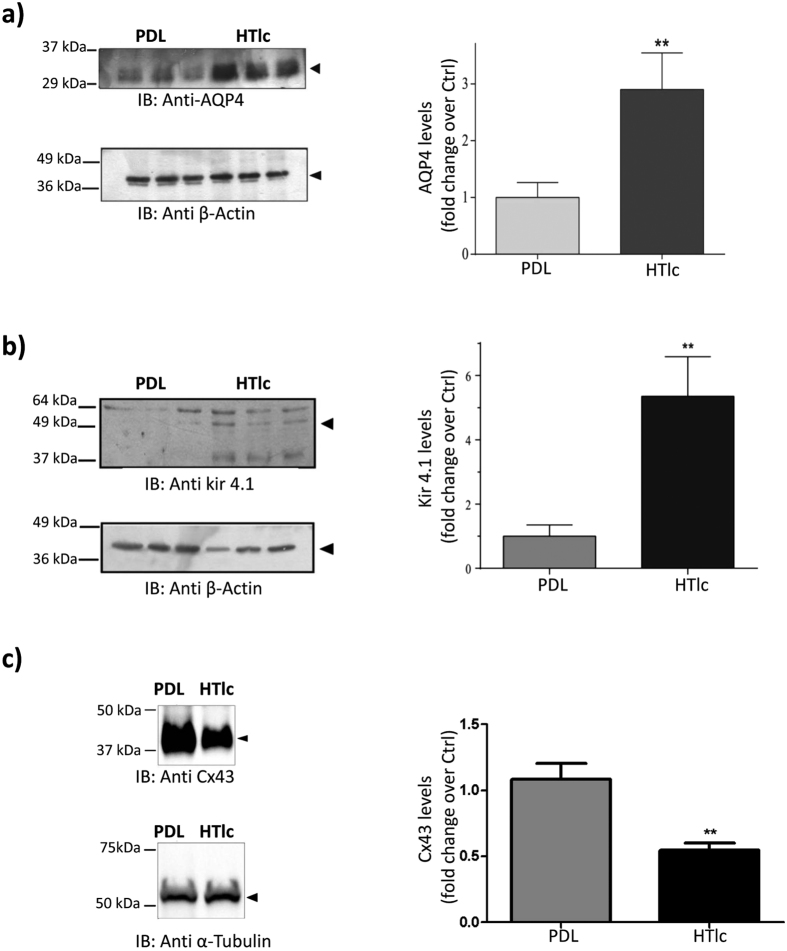
Western blot analyses and quantification of protein expression of AQP4 (**a**) Kir4.1 (**b**) and Cx43 (**c**) on astrocytes plated on PDL and HTlc. β-actin (**a,b**, lower panels) and α-tubulin were re-blotted and used as controls for signal normalization. Histogram plots evidencing change in protein expression with respect to the control are reported in the right panels of the figures. Values are the mean ± SE. (n = 3; *p < 0.01). Cropped images of the entire blots are reported. However, no other specific signals were observed within the blot length.

Western blot analyses and relative quantification (Fig. 8) revealed that the expression levels of AQP4 (Fig. 8a) and Kir4.1 (Fig. 8b) were significantly higher in cells grown on HTlc. Notably, with respect to Kir4.1 the band that was highly expressed in cells grown on HTlc was the one at 50 KDa (Fig. 8b) that corresponded to the band expressed in the plasma membrane in astrocytes^22^.

Previous studies suggested that Connexin 43 (Cx43) could have molecular or functional interaction with AQP4^63^. Thus, we also evaluated the expression of Cx43 that was lower in HTlc compared to PDL plated cells (Fig. 8c). However, the expression of Cx43 was lower on cells grown on HTlc compared to those grown on PDL,

Collectively these results indicate that growth of astrocytes on HTlc induced up-regulation of AQP4 and Kir4.1 protein expression and patterned distributions of these proteins in the plasma membrane of astroglial endfeet.

It might be asked whether the up-regulated protein and membrane expression of AQP4 and Kir4.1 might be causal or a secondary effect of mophological cell remodelling. It is plausible that astrocytes sensing the different topography underneath might need to rearrange their volume distribution and spatial buffering capability. In a previous study Nicchia and colleagues showed that the astroglial cells can change their morphology independently of AQP4 expression, and thus AQP4 plasma membrane localization is likely to be secondary to actin cytoskeleton remodelling and strictly linked to cell morphology^25^.

It has been shown that the use of a nanopatterned surface to culture human neural stem cells (hNSC) can induce direct hNSC differentiation toward neurons rather than astrocytes, by acting on the cytoskeleton and by promoting Na^+^ channel current and protein expression in the plasma membrane. This study indicates that the dimensions and topography of nanostructures are mechanical and biophysical parameters that are important to promote or suppress a specific neural/glial cell phenotype^60^. Moreover, a recent model developed by Camassa *et al.*, showed that the highly specialized differentiated astrocytes can be established *in vitro* through inductive processes that include mechanical factors and cytoskeleton remodelling^31^. In this view and in line with these studies, our results indicate that the rearrangement of the actin cytoskeleton and of focal adhesion points are responsible for the induction of morphological differentiation and the marked molecular up-regulation of AQP4 and Kir4.1 observed in cells grown on HTlc films. Accordingly, the lower expression of Cx43 might depend on the lower number of interaction points between cells due to the increased differentiation process.

One of the main goals in this study was to verify the effects of HTlc on astroglial K^+^ channels and AQP4 function that are known to be involved in the physiology and pathophysiology of CNS *in vivo*.

**Figure 9:**
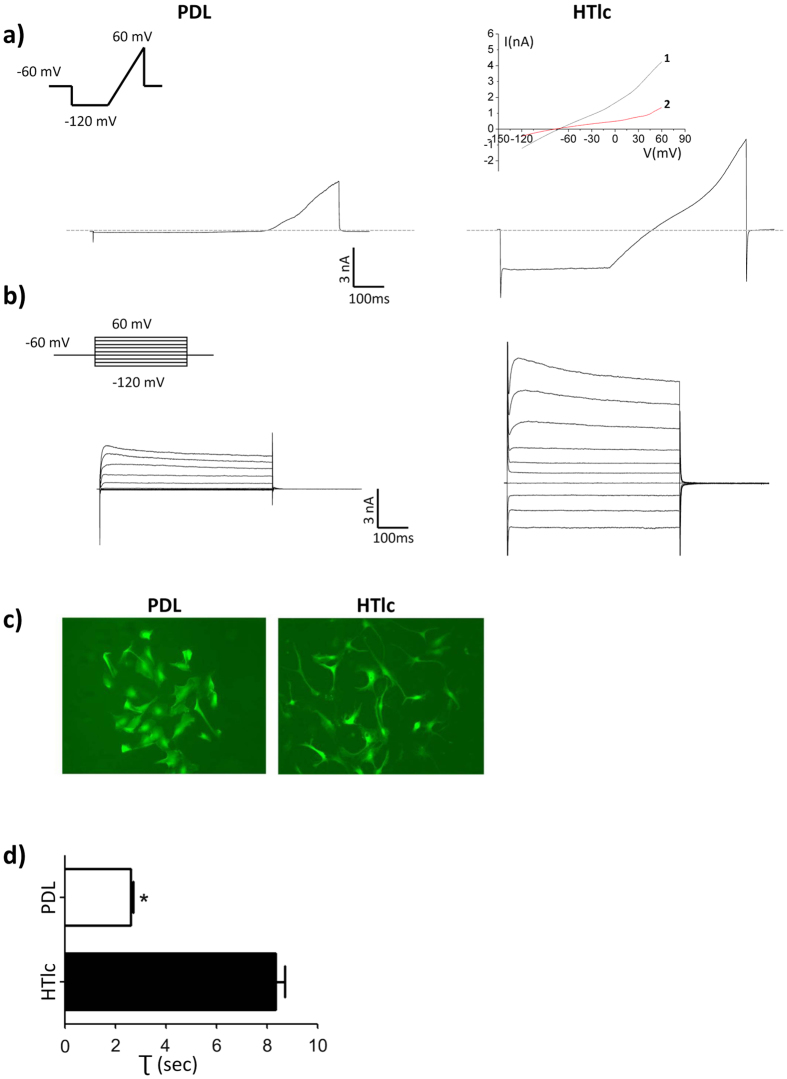
Functional properties of HTlc-plated astrocytes. (**a,b**) Typical current traces evoked by a holding potential (Vh) of −60 mV stimulating astrocytes with a voltage ramp or voltage step family (insets). Astrocytes (left panels) plated on PDL displayed only voltage-dependent outward rectifying K^+^ conductance. Astrocytes plated on HTlc coated coverslips (Right panels), display an inward conductance in response to a hyperpolarizing stimulus that is inhibited after extracellular superfusion with Ba^2+^ (**a**, inset, red trace 2). Note inhibition by Ba^2+^ of outward currents in HTLc-treated astrocytes, suggesting a weak rectification profile of the inward current typical of Kir4.1. (**c**) Micrograph of calcein-loaded astrocytes showing the different shape of astrocytes grown on PDL vs those grown on HTlc. Histogram summarizing the swelling rates (τ) for the astrocytes grown on PDL or on HTlc. Value is the mean ± SE. (n = 30; P < 0.05).

To this end, whole-cell patch-clamp and water permeability measurements were performed on cells at low density, 3 days after replating on PDL and HTlc (Fig. 9).

With intracellular and extracellular saline controls, cells were voltage clamped at a holding potential (V_h_) of −60 mV and, after stepping to −120 mV for 400 ms, a slow ramp (180 mV/600 ms) from −120 to 60 mV (inset in Fig. 8a) was applied to evoke whole-cell currents. The current amplitude was recorded and passive membrane properties were calculated (Table 2). The resting membrane potentials (V_mem_) of PDL-seeded cells were −36 ± 3 mV, which was comparable with values previously reported for primary astrocytes grown *in vitro* on different substrates^22^,^23^. V_mem_ was more hyperpolarized (~−70 mV) in HTlc-plated cells compared to PDL plated cells. Accordingly, a significant decrease was observed in the input resistance values (726 ± 94 MΩ in PDL-astrocytes, 55 ± 16 MΩ in cells grown on HTlc) while the specific conductance (spG; 0.58 ± 0.01 ns/pF for HTlc-plated cells and 0.04 ± 0.01 for PDL) recorded at −60 mV was one order of magnitude higher in HTlc-plated astrocytes than in PDL plated cells. As an additional indication that HTlc films modify astroglial cell morphology in cells seeded at low density, the capacitance was significantly higher in HTlc plated cells than in PDL plated cells (48 ± 6 pF for PDL-plated astrocytes; 61 ± 7 pF for HTlc plated cells).

The typical current profile of PDL and HTlc plated astrocytes, in response to the applied voltage ramp protocol, is shown in Fig. 9a. The ramp current traces in PDL-plated cells displayed a strong outward rectification, as witnessed by negligible currents recorded at membrane potentials below −40 mV (Fig. 9a, left pannel). Compared to currents recorded on the same days in PDL-plated cells, the current traces elicited in HTlc- astrocytes (Fig. 9a, right panel), showed a double rectification profile with large inward currents activated at membrane potentials more negative than −40 mV. Importantly, current density recorded at −120 mV and +60 mV was significantly higher for HTlc compared to PDL-plated cells (-3.6 ± 0.6 pA/pF at −120 mV and 44 ± 5.1 at +60 mV for PDL (n = 20); −27 ± 4 pA/pF at −120 mV and 75 ± 10.5 at +60 mV for HTlc-plated cells (n = 27)). It has previously been shown that barium (Ba^2+^) selectively blocks Kir channels in cultured astrocytes^12^,^22^,^23^,^64^. The inward current expressed by HTlc plated cells was inhibited by sub-milli-molar concentrations (200 μM) of Ba^2+^, as indicated by the IV plot in the inset of Fig. 9a. Note that a part of the outward current was also sensitive to Ba^2+^ application, suggesting a weak-rectifying nature of the Kir conductance, resembling Kir4.1 conductance previously reported in differentiated astrocytes *in vitro* and *in situ*^12^,^22^,^23^,^24^,^64^.

To analyze the voltage- and time-dependencies of conductance expressed by PDL- and HTlc-plated cells, astrocytes were stimulated with 500-ms voltage steps (V_h_ = −60 mV) from −120 to 60 mV in 20 mV increments (inset of Fig. 9b). Representative current traces obtained on PDL and HTlc astrocytes in response to the applied voltage are displayed in Fig. 8b. Rapidly activating, non-inactivating, voltage-dependent whole-cell currents were elicited with the family of voltage-step protocols at potentials positive to −40 mV in PDL astrocytes (Fig. 9b left panel). Differently, in response to a voltage-step protocol, inward currents at negative membrane potentials were induced in HTlc plated cells (Fig. 9b, right panel), which were fully activated within 50 ms and did not display any time-dependent inactivation. All the data indicate that PDL-plated astrocytes have voltage-gated K^+^ channels activated at membrane potentials more positive than −40 mV. The biophysical properties recorded on PDL plated cells overlap those of K_DR_ previously characterized *in vitro* on different substrates such as polystyrene Petri dishes, or poly-ornitine and silk fibroin^23^,^24^,^25^. Furthermore, Kir conductance is responsible for mediating the up-regulated conductance in HTlc-plated cells with biophysical and pharmacological features of Kir4.1 previously reported for astrocytes differentiated *in vitro* and *in situ*^12^,^22^,^23^,^24^,^64^.

Finally, water permeability was measured using the calcein method (Fig. 9c, upper panel)^65^,^66^. The histogram plot reported in Fig. 9d summarises the swelling rates (τ) for the astrocyte grown on PDL or on HTlc, indicating that the swelling rate in response to solution exchange between saline and hypotonic solution was higher in astrocytes grown on HTlc than that of those grown on PDL.

Different methods have been recently reported to promote astrocytic functional differentiation via addition of chemicals to the bath media^22^,^23^,^24^,^25^. Of note among them it has been shown that, by using agents acting on the actin cytoskeleton, the expression and function of AQP4 and Kir 4.1 can be remodelled *in vitro*^22^,^23^,^24^,^25^,^66^. This is not surprising as extracellular soluble signals are known to play a critical role in maintaining astrocytes homeostasis in the CNS^1^,^3^. However, the CNS is also composed of extracellular matrix macro-molecules that interact and mechanically support astroglial cells, by acting also via physical parameters. In this respect, the use of nanoscale HTlc could mimic the interaction of astrocytes with components of the extracellular matrix^31^.

It has been shown that functionalized single-walled carbon nanotube films induce morphological and functional changes such as increased glutamate uptake in mouse cortical astrocytes^28^,^29^,^67^. Moreover, it has been recently discovered that nano-roughness of silica films can modulate the function of hippocampal neurons and their relationship with astrocytes via mechanical stimuli^31^. Finally, different models of Blood Brain Barrier have been recently demonstrated using pore filters^31^ as well as a combination of filters and functionalized multi-walled carbon nanotubes^68^.

However, with respect to previous models our study is the first one reporting the demonstration of functional and molecular up-regulation of both Kir4.1 and AQP4 *in vitro* in astrocytes, which are essential and pivotal molecules in astroglial homeostatic microdomains *in vivo*^1^,^2^,^3^,^4^,^5^,^6^,^7^,^8^. Moreover, our approach has the advantage that we could obtain astroglial differentiation by using casted films without any need of either patterning or chemical functionalization of the surface.

## Conclusions

We have demonstrated that a new simple and ready to use substrate promotes astrocyte molecular and functional differentiation *in vitro* without needing to add chemicals to the bath media.

The HTlc film enables viability and growth of astrocytes even after weeks of culture. The mechanism underneath the differentiation involves the focal adhesion complex and the actin cytoskeleton rearrangement. Our results open the route for the potential use of HTlc as a substrate for the growth and the study of differentiated astrocytes *in vitro* without needing to nanopattern bio-interfaces, or chemical functionalization of the surface.

The present model might be an important tool to promote an increase in knowledge of the molecular events and functional dynamics that have clinical relevance in several cerebral disorders and that involve dishomeostasis of the CNS, through alteration of the expression of AQP4 and Kir4.1 at the plasma membrane of astroglial foot-processes.

## Methods

### Synthesis of ZnAl-HTlc nanoparticles and film preparation

Colloidal aqueous dispersion of ZnAl-HTlc nanoparticles having the formula [Zn_0.72_Al_0.28_(OH)_2_]Br_0.28_ 0.69 H_2_O were prepared by the double-microemulsion technique described in the refs 51 and 52. The chemical composition of the ZnAl-HTlc was determined by TG/DTA and ICP-OES analyses. TEM and AFM studies revealed that the products of the synthesis were nanoparticles 150–200 nm in width and 20–30 nm high^51^. For the film preparation, a 160 μL aliquot of ZnAl-HTlc colloidal dispersion at a concentration of 1 mg/mL was dropped on 19 mm diameter glass coverslips and successively dried for 4 h in a sterile hood. The obtained ZnAl-HTlc films were used for transmittance, AFM, SEM, XRD, cell viability and immunofluorescence measurements. In this manuscript, the ZnAl-HTlc films will be referred to simply as HTlc. Microparticles of ZnAl-HTlc were obtained using the urea method reported in ref. 69.

### Film characterization

Transmittance spectra were collected using a Jasco V-550 UV-Vis Spectrophotometer. XRD patterns were taken with a Philips X’PERT PRO MPD diffractometer operating at 40 kV and 40 mA, with a step size of 0.0170 2*θ* degree, and a step scan of 20 s, using Cu Kα radiation and an X’Celerator detector. Atomic Force Microscope (AFM) topographical images were collected using an NT-MDT Solver Scanning Probe Microscope operated in tapping mode.

### Cell culture

The experiments were performed according to the Italian law on protection of laboratory animals, with the approval of a local bioethical committee of the University of Bologna Department of Pharmacy and Biotechnology and under the supervision of the veterinary commission for animal care and comfort at the University of Bologna. Every effort was made to minimize the number of animals used and their suffering.

Primary cultures of pure cortical rat astrocytes were prepared as described previously^22^,^23^,^24^. Briefly, cerebral cortices devoid of meninges from postnatal rats (p0-p2) were triturated and placed in cell culture flasks containing Dulbecco’s modified Eagle’s medium (DMEM)–Glutamax added with 15% fetal bovine serum (FBS) and penicillin–streptomycin (100 U/mL and 100 lg/mL, respectively) (Gibco-Invitrogen, Milan, Italy). Cells were maintained in culture flasks in a humidified incubator with 5% CO_2_ for 2–5 weeks. When confluence was reached, astrocytes were dispersed using trypsin–EDTA 0.25%, and the cell suspension was re-plated on PDL or HTlc-coated glass coverslips (19 mm diameter) at a density of 7–10 × 10^3^ per dish.

### Cell viability and counting

The time course of astrocyte viability and the biocompatibility on PDL and HTLc films were analyzed by means of AlamarBlue (AB) assay, as described previously^70^,^71^.

On the day of the assay, AB reagent was added directly to the culture medium (10% v/v) of each sample. After 6 h of incubation, 100 μl of AB-containing medium was transferred into a 96 multiwell-plate and fluorescence measurement (545 nm_Ex_/590 nm_Em_) was performed using a Thermo Scientific Varioskan Flash Multimode Plate Reader.

On the same day, cells from the same preparation were stained with DAPI at different time points. Fifteen different fields of 0.6 × 0.6 mm were captured for each sample, using a Nikon ECLIPSE 80i microscope, equipped with appropriate fluorescence filters and a Nis-elements F300 CCD camera at each time point. Nuclei were counted and the number of cells/mm^2^ was calculated and expressed as mean ± SE.

### Cell differentiation measurement

Differentiation induced by interfacing astrocytes with HTLc film, was evaluated according to the following morphological criterion: elongated and star-like astrocytes were distinguished from polygonal astrocytes, considered as undifferentiated cells^72^,^73^. In particular, a cell was considered differentiated when the vertical axis of each cell was at least two times longer than the horizontal axis. Cell imaging and morphological analyses were performed by FDA assay, as described previously^23^,^74^. Briefly, astrocytes plated on the different coverslips were mounted on a custom made perfusion chamber and incubated for 5 min with fluorescein diacetate (Sigma Aldrich). Samples were rinsed with PBS three times. A sequence of confocal images (10 to 15 different fields of 0.6 × 0.6 mm for each sample) was taken using a Nikon TE 2000 inverted confocal microscope (20× oil-objective). Living cells were counted and the number of cells/mm^2^ was calculated and compared at each analyzed time point.

Quantitative analysis was carried out by counting differentiated and undifferentiated cells from astrocytes in FDA-positive (viable) cells plated on PDL and HTLc film and imaged after 3 div and 7 div. The number of differentiated cells was expressed as a percentage of the total number of live cells.

### Immunofluorescence and Confocal Microscopy

The following antibodies were used: Mouse anti-GFAP (Sigma Aldrich, Milan, Italy), Goat anti-AQP4 polyclonal (Santa Cruz Biotechnology, Santa Cruz, CA), rabbit anti-Kir 4.1 (Alomone, Jerusalem, Israel) all diluted to 1:300 and Rabbit anti-Vinculin (Life Techonogies, Monza, Italy) diluted 1:200 were used as primary antibodies. The secondary antibodies used for immunofluorescence were donkey anti-mouse CY3 (Jackson Labs), Alexa488-conjugated donkey anti-rabbit and Alexa594-conjugated donkey anti-goat (Molecular Probes, Eugene, OR) at a dilution of 1:1000. Cultured astrocytes plated on coverslips were fixed in 4% paraformaldehyde, washed in phosphate buffered saline (PBS), and permeabilized with 0.3% Triton X-100 in PBS. After blocking with 1% BSA in PBS, cells were incubated with primary antibodies for 2 h at RT, washed in PBS and incubated for 1 h at RT with Alexa conjugated secondary antibodies and with ATTO 647N-Phalloidin (Fluka Analytical, St Louis, MO) and Phalloidin-FITC (Sigma-Aldrich, Milan, Italy) to stain F-actin filaments. Coverslips were mounted on slides, using a mounting medium (50% Glycerol, 0.01% N-Propil-Gallate in PBS) and examined with a confocal microscope (TCS SP5, Leica) or with a Nikon TE 2000 inverted confocal microscope equipped with 20× and 60× oil-objectives and a 400 nm diode, 488 nm Ar^+^ and 543 nm He-Ne lasers as exciting sources. Micrometric images of Vinculin-stained cells were captured with a Nikon Eclipse 80i fluorescent microscope equipped with 20× and 40× objectives.

### Western Blot analyses

Western blot analyses was performed as described previously^22^. Briefly, samples containing 25 μg total protein were separated on SDS–polyacrylamide gels and electrotransferred on to nitrocellulose membranes (Bio-Rad Laboratories), blocked in 5% BSA in PBS containing 0.05% Tween 20 and probed overnight at 4 °C with primary antibodies against Kir4.1 (1:400, Alomone Laboratories, Jerusalem, Israel), AQP4 (1:500, Santa Cruz Biotechnology) and Cx43 (1:500, Sigma Aldrich). After washing with phosphate-buffered saline plus Tween 0.05% v/v (PBST) membranes were incubated with horseradish peroxidase-conjugated IgG secondary antibodies (Sigma) and developed with ECL-Plus (Bio-Rad).

### Scanning Electron Microscopy

Astrocytes plated on HTlc films and PDL were fixed with 2.5% glutaraldehyde in phosphate buffer saline (PBS) at 4 °C, for 1 hour. Each sample was than rinsed three times in PBS for 5 minutes before being stained for 1 hour in 1% osmium tetra-oxide (O_s_O_4_) at room temperature; three further rinsings with distilled water were then performed. Then samples were sequentially dehydrated in 50%, 75%, 95% and 99% ethanol. Dried specimens were sputter-coated (QR150RS, Quorum Technologies Ltd, East Sussex, UK) with gold (10 nm thickness) before analysis with a SEM-FEG Hitachi S4000.

### Electrophysiology

Current recordings were obtained with the whole-cell configuration of the patch-clamp technique. Patch pipettes were prepared from thin-walled borosilicate glass capillaries to have a tip resistance of 2–4 MΩ when filled with the standard internal solution. Membrane currents were amplified (List EPC-7) and stored on a computer for off-line analysis (pClamp 6, Axon Instrument and Origin 6.0, MicroCal). Because of the large current amplitude, the access resistance (below 10 MΩ) was corrected by 70–90%. Experiments were carried out at room temperature (20–24 °C).

### Solutions and chemicals

All salts and chemicals employed for the investigations were of the highest purity grade (Sigma). For electrophysiological experiments, the standard bath saline was (mM): 140 NaCl, 4 KCl, 2 MgCl_2_, 2 CaCl_2_, 10 HEPES, 5 glucose, pH 7.4 with NaOH and osmolarity adjusted to ∼315 mOsm with mannitol. The intracellular (pipette) solution was composed of (mM): 144 KCl, 2 MgCl_2_, 5 EGTA, 10 HEPES, pH 7.2 with KOH and osmolarity ∼300 mOsm. When using external solutions with different ionic compositions, salts were replaced equimolarly. The different salines containing pharmacological agents were applied using a gravity-driven, local perfusion system at a flow rate of ∼200 μl/min positioned within ∼100 μm of the recorded cell.

### Osmotic Water Permeability

Primary cultured astrocytes grown on 20 mm diameter round glass coverslips were washed with PBS and incubated with 10 μM of Calcein-AM (Invitrogen, Milan, Italy) for 25 min at RT. The osmotic properties of astrocytes were analysed using the time course of fluorescence quenching methods^65^, measured in response to the osmotic gradient. Water permeability was measured by using a Nikon TE2000U Microscope equipped with a Nikon Laser TIRF setup^75^. Cells were initially perfused with PBS (30 mL/min, temperature 10 °C) and then subjected to hypoosmotic treatment by reducing the NaCl concentration of PBS. The time course of fluorescence, measured in response to osmotic gradient, was used to assess the osmotic properties of cells. The kinetics of osmotic volume changes were characterized by comparing the time constants of cell swelling (obtained from experimental data) fitted to a single exponential function. Images were analysed using NIS-Element AR 3.1 software.

### Statistical Analysis

In the histogram plots, the values are presented as means ± SE of the number of experiments indicated in the figure legend. The Student’s t-test for unpaired data was used. Differences were considered significant only when P-values were <0.05.

## Additional Information

**How to cite this article**: Posati, T. *et al.* A Nanoscale Interface Promoting Molecular and Functional Differentiation of Neural Cells. *Sci. Rep.* **6**, 31226; doi: 10.1038/srep31226 (2016).

## Supplementary Material

Supplementary Information

## Figures and Tables

**Table 1:** Percentage of differentiated astrocytes grown on PDL and HTlc.

	3 div	7 div
PDL	21% ± 2	10% ± 3
HTlc	27% ± 4	52% ± 2

**Table 2:** Electrophysiological properties of astrocytes plated on PDL and HTlc 1.2.

Electrophysiological properties	V_mem_ (mV)	Cp (pF)	I R (MΩ)	spG (nS/pF)	I _−120mV_ (pA/pF)	I _60mV_ (pA/pF)
PDL	−36 ± 3	48 ± 6	726 ± 94	0.04 ± 0.01	−3.6 ± 0.6	44 ± 5.1
HTlc	−70 ± 1**	61 ± 6	55 ± 16**	0.58 ± 0.1**	−27 ± 4**	75 ± 10.5*

Mean values of electrophysiological membrane properties of astrocytes grown on PDL (n = 16) and HTlc (n = 20). V_mem_: resting membrane potential; Cp:membrane capacitance; IR: input resistance; spG: specific conductance; I_−120mV_: inward current density; I_60mV_: outward current density. *p < 0.05 **p < 0.01 (Student’s t-test).

## References

[b1] Nedergaard M., Ransom B. & Goldman S. A. New roles for astrocytes: redefining the functional architecture of the brain. Trends Neurosci. 26, 523–530 (2003). 14522144 10.1016/j.tins.2003.08.008

[b2] Benfenati V. & Ferroni S. Water transport between CNS compartments: functional and molecular interactions between aquaporins and ion channels. Neuroscience 168, 926–940 (2010). 20026249 10.1016/j.neuroscience.2009.12.017

[b3] Benfenati V. & Ferroni S. In Microdynamics of Water and Ion Homeostasis in the Brain: Role of Aquaporins and Ion Channels of Astroglial Cells; in Homeostatic control of brain function ; (eds Boison D. & Masino S. A.); First Edition, Oxford University Press (2015); “in press”.

[b4] Kimelberg H. K. Astrocytic swelling in cerebral ischemia as a possible cause of injury and target for therapy. Glia 50, 389–397 (2005). 15846797 10.1002/glia.20174

[b5] Steinhäuser C., Grunnet M. & Carmignoto G. Crucial role of astrocytes in temporal lobe epilepsy. Neuroscience, doi: 10.1016/j.neuroscience.2014.12.047 (2015). 25592426

[b6] Yang J. *et al.* Loss of astrocyte polarization in the tg-ArcSwe mouse model of Alzheimer’s disease. J Alzheimers Dis. 27, 711–722 (2011). 21891870 10.3233/JAD-2011-110725

[b7] Verkhratsky A. & Steinhäuser C. Ion channels in glial cells. Brain Res. Rev. 32, 380–412 (2000). 10760549 10.1016/s0165-0173(99)00093-4

[b8] Kimelberg H. K., Macvicar B. A. & Sontheimer H. Anion channels in astrocytes: biophysics, pharmacology and function. Glia 54, 747–757 (2006). 17006903 10.1002/glia.20423 PMC2556042

[b9] Orkand R. K., Nicholls J. G. & Kuffler S. W. Effect of nerve impulses on the membrane potential of glial cells in the central nervous system of amphibia. J. Neurophysiol. 29, 788–806 (1966). 5966435 10.1152/jn.1966.29.4.788

[b10] Newman E. A., Frambach D. A. & Odette L. L. Control of extracellular potassium levels by retinal glial cell K^+^ siphoning. Science 225, 1174–1175 (1984). 6474173 10.1126/science.6474173 PMC2693189

[b11] Heuser K. *et al.* Loss of perivascular Kir4.1 potassium channels in the sclerotic hippocampus of patients with mesial temporal lobe epilepsy. J Neuropathol Exp Neurol. 71, 814–25 (2012). 22878665 10.1097/NEN.0b013e318267b5af PMC3470834

[b12] Bockenhauer D. *et al.* Epilepsy, ataxia, sensorineural deafness, tubulopathy, and KCNJ10 mutations. N Engl J Med. 360, 1960–1970 (2009). 19420365 10.1056/NEJMoa0810276 PMC3398803

[b13] Scholl U. I. *et al.* Seizures, sensorineural deafness, ataxia, mental retardation, and electrolyte imbalance (SeSAME syndrome) caused by mutations in KCNJ10. Proc Natl Acad Sci. 106, 5842–5847 (2009). 19289823 10.1073/pnas.0901749106 PMC2656559

[b14] Olsen M. L. & Sontheimer H. Mislocalization of Kir channels in malignant glia. Glia 46, 63–73 (2004). 14999814 10.1002/glia.10346 PMC2548404

[b15] Tan G., Sun S. Q. & Yuan D. L. Expression of Kir 4.1 in human astrocytic tumors: correlation with pathologic grade. Biochem Biophys Res Commun. 367, 743–747 (2008). 18191638 10.1016/j.bbrc.2008.01.014

[b16] Nagelhus E. A. *et al.* Immunogold evidence suggests that coupling of K^+^-siphoning and water transport in rat retinal Müller cells is mediated by a coenrichment of Kir4.1 and AQP4 in specific membrane domains. Glia 26, 47–54 (1999). 10088671 10.1002/(sici)1098-1136(199903)26:1<47::aid-glia5>3.0.co;2-5

[b17] Nagelhus E. A., Mathiisen T. M. & Ottersen O. P. Aquaporin-4 in the central nervous system: cellular and subcellular distribution and coexpression with Kir4.1. Neuroscience 129, 905–913 (2004). 15561407 10.1016/j.neuroscience.2004.08.053

[b18] Amiry-Moghaddam M. & Ottersen O. P. The molecular basis of water transport in the brain. Nat Rev Neurosci 4, 991–1001 (2003). 14682361 10.1038/nrn1252

[b19] Frigeri A., Nicchia G. P. & Svelto M. Aquaporins as targets for drug discovery. Curr Pharm Des. 13, 2421–2427 (2007). 17692010 10.2174/138161207781368738

[b20] Olsen M. L. & Sontheimer H. J. Functional implication for Kir 4.1. channels in glial biology: from K^+^ buffering to cell differentiation. Neurochem. 107, 589–601 (2008). 10.1111/j.1471-4159.2008.05615.x PMC2581639 18691387

[b21] Sontheimer H. An unexpected role for ion channels in brain tumor metastasis. Exp. Biol. Med. 233, 779–791 (2008). 10.3181/0711-MR-308 PMC2557067 18445774

[b22] Benfenati V., Caprini M., Nobile M., Rapisarda C. & Ferroni S. Guanosine promotes the up-regulation of inward rectifier potassium current mediated by Kir4.1 in cultured rat cortical astrocytes. J Neurochem. 98, 430–345 (2006). 16805837 10.1111/j.1471-4159.2006.03877.x

[b23] Benfenati V. *et al.* A silk platform that enables electrophysiology and targeted drug delivery in brain astroglial cells. Biomaterials 31, 7883–7891 (2010). 20688390 10.1016/j.biomaterials.2010.07.013 PMC2966966

[b24] Ferroni S., Marchini C., Schubert P. & Rapisarda C. Two distinct inwardly rectifying conductances are expressed in long term dibutyryl-cyclic-AMP treated rat cultured cortical astrocytes. FEBS Lett. 367, 319–325 (1995). 7607331 10.1016/0014-5793(95)00588-z

[b25] Nicchia G. P. Actin cytoskeleton remodeling governs aquaporin-4 localization in astrocytes. Glia 56, 1755–1766 (2008). 18649401 10.1002/glia.20724

[b26] Isaksson J. *et al.* Electronic control of Ca2^+^ signalling in neuronal cells using an organic electronic ion pump. Nat Mater. 6, 673–679 (2007). 17643105 10.1038/nmat1963

[b27] Benfenati V. *et al.* Photostimulation of whole-cell conductance in primary rat neocortical astrocytes mediated by organic semiconducting thin films. Adv Healthc Mater. 3, 392–399 (2014). 23966220 10.1002/adhm.201300179

[b28] Gottipati M. K., Kalinina I., Bekyarova E., Haddon R. C. & Parpura V. Chemically functionalized water-soluble single-walled carbon nanotubes modulate morpho-functional characteristics of astrocytes. Nano Lett. 12, 4742–4747 (2012). 22924813 10.1021/nl302178s

[b29] Gottipati M. K. *et al.* Chemically functionalized single-walled carbon nanotube films modulate the morpho-functional and proliferative characteristics of astrocytes. Nano Lett. 13, 4387–4392 (2013). 23937522 10.1021/nl402226z

[b30] Gottipati M. K., Bekyarova E., Haddon R. C. & Parpura V. Chemically functionalized single-walled carbon nanotubes enhance the glutamate uptake characteristics of mouse cortical astrocytes. Amino Acids. 47, 1379–1388 (2015). 25837300 10.1007/s00726-015-1970-9 PMC4459931

[b31] Camassa L. M. *et al.* Mechanisms underlying AQP4 accumulation in astrocyte endfeet. Glia, doi: 10.1002/glia.22878 (2015). 26119521

[b32] Blumenthal N. R., Hermanson O., Heimrich B. & Shastri V. P. Stochastic nanoroughness modulates neuron-astrocyte interactions and function via mechanosensing cation channels. Proc Natl Acad Sci USA 111, 16124–16129 (2014). 25349433 10.1073/pnas.1412740111 PMC4234571

[b33] Rives V. In Layered Double Hydroxides: Present and Future (Nova Science Publishers, 2001).

[b34] Khan A. I. & O’Hare D. J. Intercalation chemistry of layered double hydroxides: recent developments and applications. Mater. Chem. 12, 3191−3198 (2002).

[b35] Leroux F. & Taviot-Guého C. J. Fine tuning between organic and inorganic host structure: new trend in layered double hydroxide hybrid assemblies. J Mater. Chem. 15, 3628−3642 (2005).

[b36] Posati T. *et al.* Selective internalization of Zn-Al-HTlc nanoparticles in normal and tumor cells. A study of their potential use in cellular delivery. Appl. Clay Sci. 55, 62−69 (2012).

[b37] Choi S. J., Oh J. M. & Choy J. H. Toxicological effects of inorganic nanoparticles on human lung cancer A549 cells. J. Inorg. Biochem. 103, 463–471 (2009). 19181388 10.1016/j.jinorgbio.2008.12.017

[b38] Choy J. H., Kwak S. Y., Jeong Y. J. & Park J. S. Inorganic layered double hydroxides as nonviral vectors. Angew. Chem. 39, 4042–4045 (2000). 10.1002/1521-3773(20001117)39:22<4041::aid-anie4041>3.0.co;2-c 11093198

[b39] Desigaux L. *et al.* Self-assembly and characterization of layered double hydroxide/DNA hybrids. Nano Lett. 6, 199–204 (2006). 16464034 10.1021/nl052020a

[b40] Xu Z. P. *et al.* Subcellular compartment targeting of layered double hydroxide nanoparticles. Control. Release 130, 86–94 (2008). 10.1016/j.jconrel.2008.05.021 18614254

[b41] Del Hoyo C. Layered double hydroxides and human health: An overview. Appl. Clay Sci. 36, 103–121 (2007).

[b42] Costantino U., Leroux F., Nocchetti M. & Mousty C. (ed. Bergaya F. *et al.* ) In Handbook of Clay Science, 765–791, Elsevier: Amsterdam, (2013).

[b43] Carretero M. I. & Lagaly G. Clays and Health Clays in *Pharmacy, Cosmetics, Pelotherapy, and Environmental Protection*, Special Issue of Appl. Clay Sci. Eds., vol. 36 (2007).

[b44] Rives V., Del Arco M. & Martín C. Layered Double Hydroxide-Based Nanocarriers for drug delivery. Appl. Clay Sci. 6, 88–89, 239–269 (2014).

[b45] Nocchetti M. *et al.* Ag/AgCl nanoparticle decorated layered double hydroxides: synthesis, characterization and antimicrobial properties. J. Mater. Chem. B 1, 2383–2393 (2013). 10.1039/c3tb00561e 32261073

[b46] Posati T. *et al.* Innovative multifunctional silk fibroin and hydrotalcite nanocomposites: a synergic effect of the components. Biomacromolecules 15, 158−168 (2014). 24313841 10.1021/bm401433b

[b47] Costantino U., Nocchetti M., Tammaro L. & Gorrasi G. In Multifunctional and Nanoreinforced Polymers for Food Packaging (ed. Lagaron J. M.) 43–85 (Woodhead Publishing, 2011).

[b48] Bolognesi M. *et al.* Efficiency enhancement of P3HT:PCBM solar cells containing scattering Zn-Al hydrotalcite nanoparticles in the PEDOT: PSS. Organic Photonics and Photovoltaics 1, 1–10 (2013).

[b49] Yan D. *et al.* Thin film of sulfonated zinc phthalocyanine/layered double hydroxide for achieving multiple quantum well structure and polarized luminescence. Chem. Commun. 46, 8654–8656 (2010). 10.1039/c0cc02129f 20972500

[b50] Posati T. *et al.* RSC. Adv. 4, 11840–11847 (2014).

[b51] Bellezza F., Cipiciani A., Costantino U., Nocchetti M. & Posati T. Hydrotalcite-Like nanocrystals from water in oil microemulsions. Eur. J. Inorg. Chem. 18, 2603–2611 (2009).

[b52] Bellezza F., Nocchetti M., Posati T., Giovagnoli S. & Cipiciani A. Syntesis of colloidal dispersion of NiAl, ZnAl, NiCr, ZnCr, NiFe, MgFe, hydrotalcite-like nanoparticles. J. Colloid. Interf. Sci. 376, 20–27 (2012). 10.1016/j.jcis.2012.02.034 22459023

[b53] Wang L., Li C., Liu M., Evans D. G. & Duan X. Large continuous, transparent and oriented self-supporting films of layered double hydroxides with tunable chemical composition. Chem. Commun. 2, 123–125 (2006). 10.1039/b613687g 17180220

[b54] Gardner E., Huntoon K. & Pinnavaia T. J. Direct Synthesis of Alkoxide-Intercalated Derivatives of Hydrotalcite-like Layered Double Hydroxides: Precursors for the Formation of Colloidal Layered Double Hydroxide Suspensions and Transparent Thin Films. Adv. Mater. 13, 1263–1266 (2001).

[b55] McConnell G. C. *et al.* Implanted neural electrodes cause chronic, local infiammation that is correlated with local neurodegeneration. Neural. Eng. 6, 056003–056015 (2009). 10.1088/1741-2560/6/5/056003 19700815

[b56] Kam L., Shain W., Turner J. N. & Bizios R. Selective adhesion of astrocytes to surfaces modified with immobilized peptides. Biomaterials 23, 511–515 (2002). 11761172 10.1016/s0142-9612(01)00133-8

[b57] Lin J. K., Uan J. Y., Wu C. P. & Huang H. H. Direct growth of oriented Mg-Fe layered double hydroxide (LDH) on pure Mg substrates and *in vitro* corrosion and cell adhesion testing of LDH-coated Mg samples. J. Mater. Chem. 21, 5011–5020 (2011).

[b58] Wong Y. *et al.* Efficient delivery of siRNA to cortical neurons using layered double hydroxide nanoparticles. Biomaterials 31, 8770–8779 (2010). 20709387 10.1016/j.biomaterials.2010.07.077

[b59] Nicchia G. P. *et al.* Actin cytoskeleton remodeling governs aquaporin-4 localization in astrocytes. Glia 56, 1755–1766 (2008). 18649401 10.1002/glia.20724

[b60] Yang K. *et al.* Multiscale, hierarchically patterned topography for directing human neural stem cells into functional neurons. ACS Nano. 26, 7809–7822 (2014). 25050736 10.1021/nn501182f

[b61] Peng X., Nelson E. S., Maiers J. L. & DeMali K. A. New insights into vinculin function and regulation. Int Rev Cell Mol Biol. 287, 191–231 (2011). 21414589 10.1016/B978-0-12-386043-9.00005-0 PMC4426885

[b62] Rodnight R. B. & Gottfried C. Morphological plasticity of rodent astroglia. J Neurochem. 124, 263–275 (2013). 23278277 10.1111/jnc.12087

[b63] Nicchia G. P. *et al.* New possible roles for aquaporin-4 in astrocytes: cell cytoskeleton and functional relationship with connexin43. FASEB J. 19, 1674–1676 (2005). 16103109 10.1096/fj.04-3281fje

[b64] Ransom C. B. & Sontheimer H. Biophysical and pharmacological characterization of inwardly rectifying K^+^ currents in rat spinal cord astrocytes. J Neurophysiol. 73, 333–346 (1995). 7714576 10.1152/jn.1995.73.1.333

[b65] Solenov E., Watanabe H., Manley G. T. & Verkman A. S. Sevenfold-reduced osmotic water permeability in primary astrocyte cultures from AQP-4-deficient mice, measured by a fluorescence quenching method. Am J Physiol Cell Physiol. 286, 426–432 (2004). 10.1152/ajpcell.00298.2003 14576087

[b66] Noël G. *et al.* Kir4.1 coclustering in retinal Müller glia is regulated by laminin-1 and requires the PDZ-ligand domain of Kir4.1. J Neurochem. 94, 691–702 (2005). 16033419 10.1111/j.1471-4159.2005.03191.x

[b67] Gottipati M. K., Verkhratsky A. & Parpura V. Probing astroglia with carbon nanotubes: modulation of form and function. Philos Trans R Soc Lond B Biol Sci. 369, 20130598 (2014). 25225092 10.1098/rstb.2013.0598 PMC4173284

[b68] Kafa H. *et al.* The interaction of carbon nanotubes with an *in vitro* blood-brain barrier model and mouse brain *in vivo*. Biomaterials 53, 437–452 (2015). 25890741 10.1016/j.biomaterials.2015.02.083 PMC4407899

[b69] Costantino U., Marmottini F., Nocchetti M. & Vivani R. New synthetic routes to hydrotalcytes-like compounds - characterization and properties of the obtained materials. Eur. J. Inorg. Chem. 10, 1439–1446 (1998).

[b70] Sagnella A. *et al.* Effect of different fabrication methods on the chemo-physical properties of silk fibroin films and on their interaction with neural cells. RSC Adv. 6, 9304–9314 (2016).

[b71] Sun C. *et al.* Jiang. Silver nanoparticles induced neurotoxicity through oxidative stress in rat cerebral astrocytes is distinct from the effects of silver ions. Neurotoxicology. 52, 210–21 (2016). 26702581 10.1016/j.neuro.2015.09.007

[b72] Juurlink B. H. & Hertz L. Plasticity of astrocytes in primary cultures: an experimental tool and a reason for methodological caution. Dev Neurosci. 7, 263–277 (1985). 3915290 10.1159/000112295

[b73] Swanson R. A. *et al.* Neuronal Regulation of Glutamate Transporter Subtype Expression in Astrocytes. The Journal of Neuroscience. 17, 932–940 (1997). 8994048 10.1523/JNEUROSCI.17-03-00932.1997 PMC6573161

[b74] Benfenati V. *et al.* Biofunctional silk/neuron interface. Adv. Funct. Mater. 22, 1–14 (2012).

[b75] Basco D. *et al.* AQP4-Dependent Water Transport Plays a Functional Role in Exercise-Induced Skeletal Muscle Adaptations. PLoS One 8, e58712 (2013). 23520529 10.1371/journal.pone.0058712 PMC3592820

