# Supplementary material for: A Nanoscale Interface Promoting Molecular and Functional Differentiation of Neural Cells
Source: Sci Rep. 2016 Aug 9;6:31226. doi: 10.1038/srep31226 (PMC4977496; doi:10.1038/srep31226)
Supplement: Supplementary Information [file srep31226-s1.doc]

**A Nanoscale Interface Promoting Molecular and Functional Differentiation of Neural Cells.**

Tamara Posati,^1^*^a^Assunta Pistone,^2a^ Emanuela Saracino^2^, Francesco Formaggio^2^, Maria Grazia Mola^3^, Elisabetta Troni,^1^ Anna Sagnella^4^, Morena Nocchetti^5^, Marianna Barbalinardo^2^, Francesco Valle^2^, Simone Bonetti^2^, Marco Caprini^6^, Grazia Paola Nicchia^3^, Roberto Zamboni^1^, Michele Muccini^2^ and Valentina Benfenati^1*^

1-Consiglio Nazionale delle Ricerche (CNR), Istituto per lo Sintesi Organica e la Fotoreattività (ISOF), via Gobetti, 101, 40129, Bologna, Italy.

2-Consiglio Nazionale delle Ricerche (CNR), Istituto per lo Studio dei Materiali Nanostrutturati (ISMN), via Gobetti, 101, 40129, Bologna, Italy

3-Dipartimento di Bioscienze, Biotecnologie e Biofarmaceutica, Università degli Studi di Bari “Aldo Moro”, Via Amendola 165/A, 70126, Bari, Italy

4-Laboratorio di Micro e Submicro Tecnologie abilitanti dell’Emilia-Romagna (MIST E-R), Via P. Gobetti 101, I-40129 Bologna, Italy.

5-Dipartimento di Scienze Farmaceutiche, University of Perugia, Via del Liceo 1, 06123, Perugia, Italy.

6-Department of Pharmacy and Biotechnology, via S. Donato 19/2, University of Bologna, 40127 Bologna, Italy

**Supporting Information**

Supplementary Figures:

**Figure S1:** a) Transmission of HTlc nanoplates. b) XRD patterns of HTlc films (trace b); the patterns of HTlc in powder form are also reported (trace a).


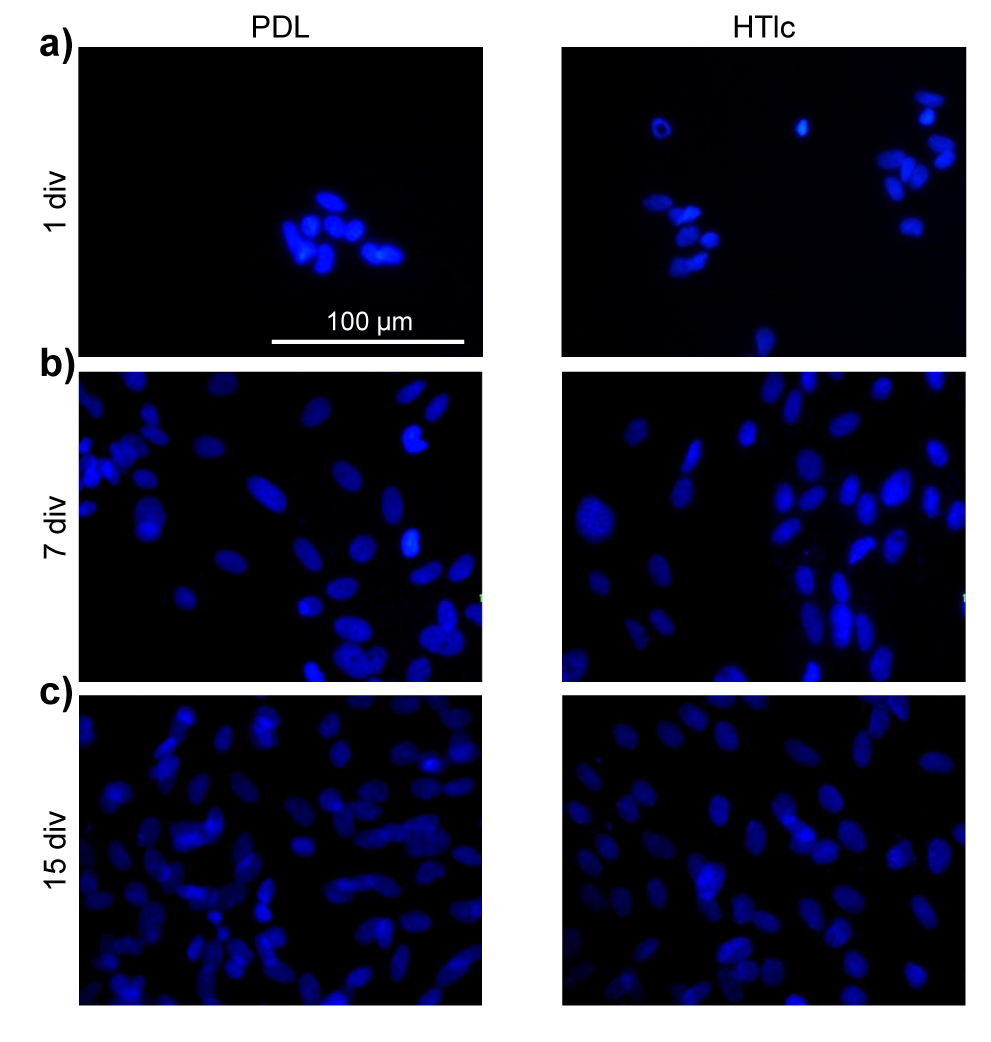


**Figure S2:**Micrographs of cell nuclei marked with DAPI in astrocytes grown on PDL (left panels) and on HTlc film (rigth panels), after 1 div (a), 7 div (b) and 15 div (c).

**Figure S3:** a) Bar plot of mean cell counts after 3 div and after 15 div of astrocytes grown on PDL (gray bar), HTlc films of nanoparticles (green bar) and HTlc substrate made by microparticles (green dashed bars). b) SEM images of HTlc microplates film.


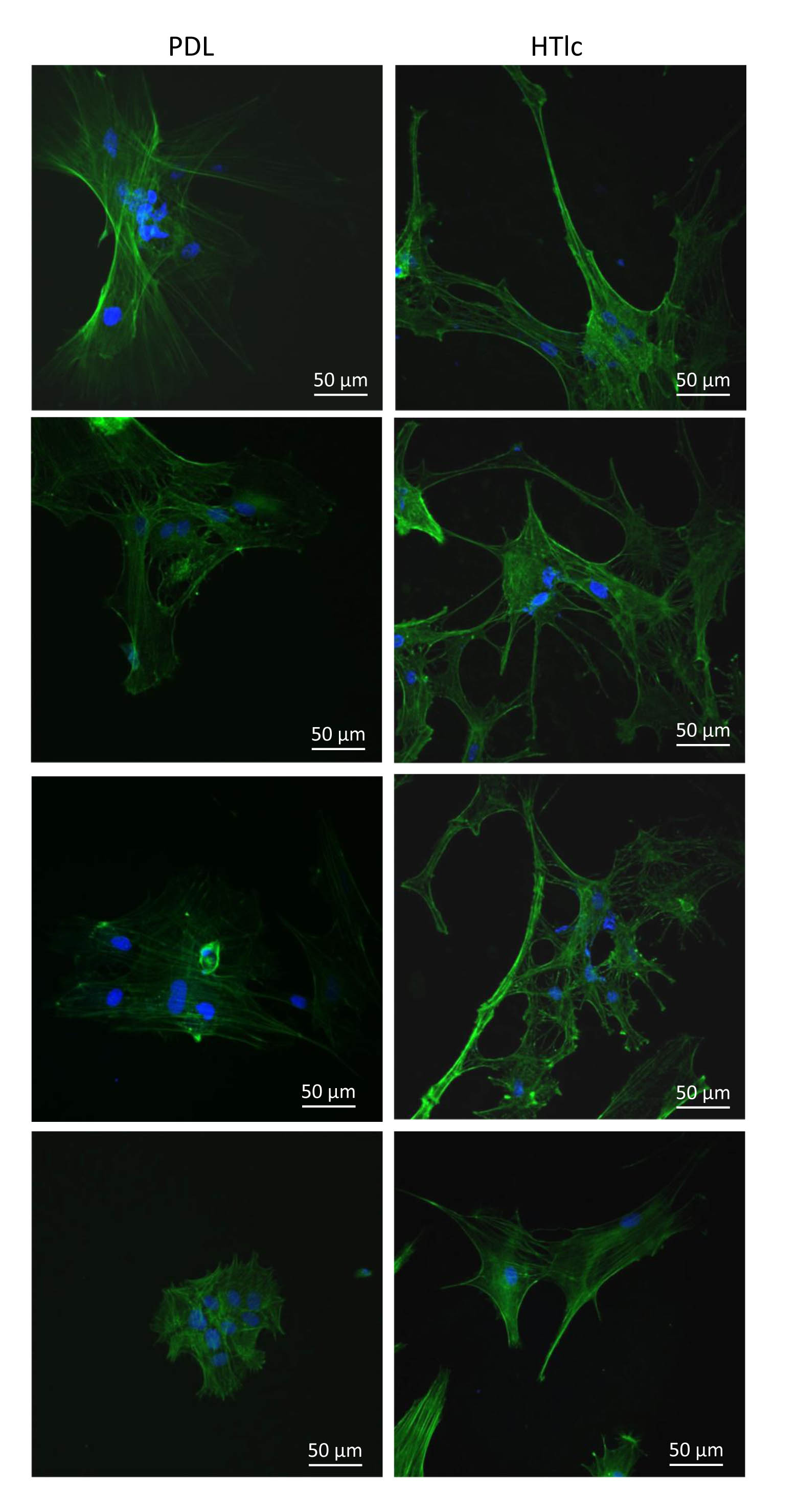


**Figure S4**:Micrographs representing astrocytes imunostained for F-actin grown on PDL (left panels) and  HTlc fims (rigth panels).
